# Transcriptome mining extends the host range of the *Flaviviridae* to non-bilaterians

**DOI:** 10.1093/ve/veac124

**Published:** 2022-12-26

**Authors:** Jonathon C O Mifsud, Vincenzo A Costa, Mary E Petrone, Ezequiel M Marzinelli, Edward C Holmes, Erin Harvey

**Affiliations:** Sydney Institute for Infectious Diseases, School of Medical Sciences, The University of Sydney, Sydney NSW 2006, Australia; Sydney Institute for Infectious Diseases, School of Medical Sciences, The University of Sydney, Sydney NSW 2006, Australia; Sydney Institute for Infectious Diseases, School of Medical Sciences, The University of Sydney, Sydney NSW 2006, Australia; School of Life and Environmental Sciences, The University of Sydney, Sydney NSW 2006, Australia; Sydney Institute of Marine Science, 19 Chowder Bay Rd, Mosman, NSW 2088, Australia; Singapore Centre for Environmental Life Sciences Engineering, Nanyang Technological University, Singapore 637551 Singapore; Sydney Institute for Infectious Diseases, School of Medical Sciences, The University of Sydney, Sydney NSW 2006, Australia; Sydney Institute for Infectious Diseases, School of Medical Sciences, The University of Sydney, Sydney NSW 2006, Australia

**Keywords:** *Flaviviridae*, *Flavivirus*, *Pestivirus*, *Hepacivirus*, virus discovery, Metazoa, phylogeny

## Abstract

The flavivirids (family *Flaviviridae*) are a group of positive-sense RNA viruses that include well-documented agents of human disease. Despite their importance and ubiquity, the timescale of flavivirid evolution is uncertain. An ancient origin, spanning millions of years, is supported by their presence in both vertebrates and invertebrates and by the identification of a flavivirus-derived endogenous viral element in the peach blossom jellyfish genome (*Craspedacusta sowerbii*, phylum *Cnidaria*), implying that the flaviviruses arose early in the evolution of the Metazoa. To date, however, no exogenous flavivirid sequences have been identified in these hosts. To help resolve the antiquity of the *Flaviviridae,* we mined publicly available transcriptome data across the Metazoa. From this, we expanded the diversity within the family through the identification of 32 novel viral sequences and extended the host range of the pestiviruses to include amphibians, reptiles, and ray-finned fish. Through co-phylogenetic analysis we found cross-species transmission to be the predominate macroevolutionary event across the non-vectored flavivirid genera (median, 68 per cent), including a cross-species transmission event between bats and rodents, although long-term virus–host co-divergence was still a regular occurrence (median, 23 per cent). Notably, we discovered flavivirus-like sequences in basal metazoan species, including the first associated with Cnidaria. This sequence formed a basal lineage to the genus *Flavivirus* and was closer to arthropod and crustacean flaviviruses than those in the tamanavirus group, which includes a variety of invertebrate and vertebrate viruses. Combined, these data attest to an ancient origin of the flaviviruses, likely close to the emergence of the metazoans 750–800 million years ago.

## 1. Introduction

The flavivirids (family *Flaviviridae)* are a group of positive-sense single-stranded RNA viruses comprising the genera *Flavivirus, Pestivirus, Pegivirus*, and *Hepacivirus.* These viruses include well-documented agents of human and livestock disease, including dengue virus, hepatitis C virus, yellow fever virus, Zika virus, and Bovine viral diarrhea virus 1. Reflecting their regular occurrence as pathogens, our understanding of flavivirid biology is necessarily skewed towards a subset of metazoan hosts, particularly those known to experience overt disease or act as reservoirs for these viruses, impeding our ability to understand the evolutionary history of this family. Currently available data suggest that all established genera, with the exception of the genus *Flavivirus*, are vertebrate-infecting viruses and do not require an arthropod vector for transmission (Simmonds et al. 2017).

The genus *Flavivirus* can itself be divided into four groups defined by phylogenetic position and host range: the (i) mosquito-borne flaviviruses, (ii) tick-borne flaviviruses, (iii) insect-specific flaviviruses, and (iv) vertebrate-specific flaviviruses, also known as the ‘no known vector’ flaviviruses (Blitvich and Firth 2017; Simmonds et al. 2017). A wide diversity of more divergent ‘flavi-like’ viruses have also been identified, including a group associated with crustaceans and decapods, as well as the tamanaviruses (after Tamana bat virus), which contains viruses from a broad range of vertebrate and invertebrate species (Price 1978; Geoghegan et al. 2018; Shi et al. 2018; Skoge et al. 2018; Parry et al. 2019; Le Lay et al. 2020; Soto et al. 2020; Costa et al. 2021). Another clade of related flavi-like viruses was recently identified in free-living parasitic flatworms (order Tricladida) (Dheilly et al. 2022).

Metagenomic surveys have identified flavivirid sequences with diverse genome structures, straying from the single 9–13 kb polyprotein that previously appeared to be canonical for the family. This expanded diversity includes a group of novel, predominantly arthropod-associated viruses—the jingmenviruses—that are both segmented and perhaps multicomponent (Qin et al. 2014; Ladner et al. 2016; Shi et al. 2016; Simmonds et al. 2017). Metagenomic studies have also expanded the host range of hepaci-, pesti- and pegiviruses in non-mammalian hosts, including the discovery of hepaci- and pegiviruses in birds (Goldberg et al. 2019; Porter et al. 2020; Chang, Rose, and Holmes 2021; Zhang et al. 2022), hepaci- and pesti-like viruses in cartilaginous fish (Chondrichthyes) (Shi et al. 2018), and hepaciviruses in reptiles and bony fish (Osteichthyes) (Shi et al. 2018; Porter et al. 2020; Costa et al. 2022; Harding et al. 2022).

The identification of flavivirid sequences in marine invertebrate and basal vertebrate lineages has led to suggestions that the evolution of the *Flaviviridae* may follow that of the metazoans through virus–host co-divergence over timescales of hundreds of millions of years (Shi et al. 2018; Bamford et al. 2022; Lensink, Yiqiao, and Lequime 2022). This, in turn, has stimulated questions regarding their host range and mode of transmission, while the complex evolutionary history of the flaviviruses and related sequences has been highlighted by their broad host range and sequence diversity. For example, the large phylogenetic gap between the cartilaginous fish and mammalian pestiviruses suggests that related viruses in bony fish, amphibians, reptiles, and birds exist but have yet to be sampled. The identification of flaviviruses in freshwater and marine crustaceans and a flavivirus-derived endogenous viral element (EVE) in the peach blossom jellyfish genome (*Craspedacusta sowerbii*, phylum *Cnidaria*) (Bamford et al. 2022) points towards an aquatic origin for the flaviviruses and highlights their long evolutionary association with the Metazoa. In particular, the cnidarian EVE suggests the existence of exogenous cnidarian flaviviruses. These are of importance for understanding the evolution of the *Flaviviridae*, as cnidarians, which include jellyfish, sea anemones, and corals, are an early branching lineage of the metazoans thought to have originated 700 million years ago (Erwin 2015). The phylogeny of the Metazoa can itself be divided into two major groups: those with bilateral body symmetry, the bilaterians, which comprise 99 per cent of all animal species, and, basal to them, the non-bilaterians, which include all the early diverging metazoan lineages—the Cnidaria, Placozoa, Porifera, and Ctenophora. Because non-bilaterians lack the body plan and circulatory system of vertebrates, it is possible that viruses in these hosts use an alternate mode of cell-to-cell transmission. To date, however, no flavivirids have been identified in these early diverging metazoan phyla.

Transcriptome mining is a proven method of virus discovery that leverages previous investment in metagenomics (Greninger 2018; Parry et al. 2019; Grimwood et al. 2021; Iwamoto et al. 2021; Miller et al. 2021; Paraskevopoulou et al. 2021; Dheilly et al. 2022; Edgar et al. 2022; Mifsud et al. 2022; Olendraite, Brown, and Firth 2022). To understand the host range of flavivirid sequences throughout the Metazoa and hence more accurately determine the age of the *Flaviviridae*, we used the Serratus RNA-dependent RNA polymerase (RdRp) search (https://www.serratus.io/explorer/rdrp) to mine the Sequence Read Archive (SRA) database for novel flavivirid sequences. To supplement this analysis, total RNA-sequencing data of the tunicate *Botrylloides leachii* was generated and screened to identify additional flavivirid sequences.

## 2. Methods

### 2.1 Screening of SRAs for flavivirid-like sequences

The Serratus RdRp search and palmID analysis suite (Babaian and Edgar 2022; Edgar et al. 2022) were used to identify datasets within the SRA (as of May 2022) that contain signatures of novel flavivirid-like sequences. This search was limited to the family *Flaviviridae* with a threshold score of ≥50 (for an explanation of the Serratus classifier score, see https://github.com/ababaian/serratus/wiki/.summary-Reports). The *de novo* transcriptome assemblies available at the National Center for Biotechnology Information (NCBI) Transcriptome Shotgun Assembly (TSA) Database (https://www.ncbi.nlm.nih.gov/genbank/tsa/) (as of June 2021) were also screened using the translated Basic Local Alignment Search Tool algorithm (TBLASTN) under default scoring parameters and the BLOSUM45 matrix. Amino acid sequences from representatives of the four *Flaviviridae* genera along with the related jingmenviruses were used as queries for the palmID and TSA database searches (Supplementary Table S1a). All novel virus sequences discovered were then used as queries in further SRA and TSA searches. The SRA and TSA search range was limited to Eukaryotes (NCBI taxonomic identifier (taxid 2759)), excluding the Viridiplantae (taxid 33090). Invertebrate datasets were limited to aquatic species as terrestrial invertebrate SRAs have been previously examined (Paraskevopoulou et al. 2021).

### 2.2 Tunicate collection, RNA extraction, and metagenomic next-generation sequencing

The tunicate *B. leachii* was collected by divers wearing surgical gloves at 0.5–3 m depth at the pier pilings in Chowder Bay, Sydney, Australia (site description in Marzinelli (2012)), on 24 November 2021. Sections of colonies were detached from the substratum using sterile tweezers, which were rinsed in 80 per cent ethanol between samples and brought to the surface, where they were placed in sterile cryogenic tubes. Samples were stored in liquid nitrogen on-site and then transferred to a −80°C freezer until extraction. Total RNA was extracted using the RNeasy Plus Mini Kit (Qiagen, Hilden, Germany) as previously described in the study by Geoghegan et al. (2021). These libraries were constructed using the Truseq Total RNA Library Preparation Protocol (Illumina). Host ribosomal RNA was depleted with the Ribo-Zero Plus Kit (Illumina), and paired-end sequencing (150 bp) was performed on the NovaSeq 6000 platform (Illlumina). Library construction and metatranscriptomic sequencing were performed by the Australian Genome Research Facility.

### 2.3 Identification of novel flavivirid genomes

Raw FASTQ files for all libraries that contained flavivirid-like sequences were obtained through the European Nucleotide Archive (https://www.ebi.ac.uk/ena/browser/home). Adapter removal and quality trimming were conducted using Trimmomatic (v0.38) with parameters SLIDINGWINDOW:4:5, LEADING:5, TRAILING:5, and MINLEN:25 (Bolger, Lohse, and Usadel 2014). To recover full-length virus sequences, raw reads were assembled *de novo* into contigs using MEGAHIT (v1.2.9) (Li et al. 2015). The assembled contigs were then compared to the NCBI non-redundant protein database (as of August 2021) and a custom *Flaviviridae* protein database using Diamond BLASTx (v2.0.9) with an *E*-value threshold of 1 × 10^−5^ (Buchfink, Xie, and Huson 2015). To identify highly divergent sequences, a custom *Flaviviridae* protein database was regularly updated with the novel viruses identified.

### 2.4 Genome extension and annotation

Sequence reads were mapped onto virus-like contigs using Bbmap (v37.98), and areas of heterogeneous coverage were manually checked using Geneious (v11.0.9) (Kearse et al. 2012; Bushnell 2014). Where possible, the extremities of contigs were manually extended and re-submitted to read mapping until the contig appeared complete or no overhanging extremities were observed. Sequences of vector origin were detected using VecScreen (https://www.ncbi.nlm.nih.gov/tools/vecscreen/) and removed. Contig abundances were calculated using the RNA-Seq by Expectation Maximization software (v1.3.0) (Li and Dewey 2011). GetORF from EMBOSS (v6.6.0) was used to predict open reading frames (ORFs) (Rice, Longden, and Bleasby 2000). To annotate protein functional domains, the InterProScan software package (v5.56) was used with the TIGRFAMs (v15.0), SFLD (v4.0), PANTHER (v15.0), SuperFamily (v1.75), PROSITE (v2022_01), CDD (v3.18), Pfam (v34.0), SMART (v7.1), PRINTS (v42.0), and CATH-Gene3D databases (v4.3.0) (Jones et al. 2014). Genome diagrams were constructed using a manually curated selection of predicted functional domains and visualized using gggenomes (Hackl and Ankenbrand, 2022).

### 2.5 Detection of endogenous virus elements

To screen for EVEs within the viral-like contigs, the putative virus-like nucleotide sequence was compared to the corresponding host genome (where available) and a subset of the whole-genome shotgun contig database (as of October 2022) using the TBLASTN algorithm with an *E*-value cutoff of 1 × 10^–20^. In addition, the virus-like sequences were checked for host gene contamination using the contamination function implemented in CheckV (v0.8.1) (Nayfach et al. 2021). All EVEs were removed from subsequent analyses.

### 2.6 Assessment of library composition

Taxonomic identification for the contigs assembled for each library was obtained by aligning them to the custom NCBI nt database using the KMA aligner and the CCMetagen program (Clausen, Aarestrup, and Lund 2018; Marcelino et al. 2020). In the case of the cigar comb jelly flavivirus, where raw reads are not publicly available, contigs from the corresponding TSA (GHXY01000001:GHXY01366104) were used as input. Virus abundance was calculated by counting the number of nucleotides matching the reference sequence with an additional correction for template length (the default parameter in KMA). Krona graphs were created using the KMA and CCMetagen methods and further edited in Adobe Illustrator (https://www.adobe.com) (Clausen, Aarestrup, and Lund 2018; Marcelino et al., 2020).

The virus sequences identified in this study were named using a combination of the host common name—if known—and the appropriate *Flaviviridae* genera (e.g. Harrimaniidae flavivirus). Virus–host assignments were made using a combination of host/virus abundance measurements and phylogenetic analyses. Where <80 per cent of host abundance was associated with the target species of the library, the possibility of alternative hosts was considered. In this case, the other organisms comprising this library were examined to determine if they might represent the source of the virus sequence. For instance, given the known host range of the flavivirids, it is more likely that these sequences are derived from metazoan species than from bacteria, fungi, or archaea. As such, metazoan species were given greater weighting when assigning putative virus–host assignments. Where host assignment proved difficult to assign with accuracy, the suffix ‘associated’ was added to the host name to signify this (e.g. digyalum oweni-associated virus). Where the taxonomic position of a virus was ambiguous, the suffix ‘-like’ was used (e.g. African cichlid flavi-like virus).

### 2.7 Phylogenetic analysis

The phylogenetic trees of the putative flavivirid sequence identified here were inferred using a maximum likelihood approach. Translated virus contigs were aligned with known flavivirid protein sequences from NCBI/GenBank using MAFFT (v7.402) employing the generalized affine gap cost algorithm (Katoh and Standley 2013; Sayers et al. 2021). Poorly aligned regions were removed using trimAl (v1.2) with a gap threshold ranging from 0.7 to 0.9 and a variable conserve value (Capella-Gutiérrez, Silla-Martínez, and Gabaldón 2009). All phylogenetic trees were estimated using IQ-TREE2. Branch support was calculated using 1,000 bootstrap replicates with the UFBoot2 algorithm and an implementation of the SH-like approximate likelihood ratio test within IQ-TREE2 (Guindon et al. 2010; Hoang et al. 2017). Selection of the best-fit model of amino acid substitution was determined using the Akaike information criterion (AIC), the corrected AIC, and the Bayesian information criterion with the ModelFinder function in IQ-TREE 2 (Kalyaanamoorthy et al. 2017; Minh et al. 2020). The trimming methods, alignment lengths, and phylogenetic models chosen in this analysis are outlined in Supplementary Table S1b. Phylogenetic trees were annotated using the R packages phytools (v1.0–3) and ggtree (v3.3.0.9) and further edited in Adobe Illustrator (https://www.adobe.com) (Revell 2012; Yu et al. 2017).

### 2.8 Assessment of cross-species virus transmission

To visualize the relative occurrence of cross-species transmission and virus–host co-divergence across the *Flaviviridae*, we analysed the co-phylogenetic relationship between viruses and their hosts. Host cladograms were created using the phyloT software, a phylogenetic tree generator based on NCBI taxonomy (http://phylot.biobyte.de/). Virus–host associations were obtained from the NCBI virus database (Brister et al. 2015; Hatcher et al. 2017) and the Virus–Host database (release 213) (Mihara et al. 2016) (accessed 14 September 2022). Tanglegrams that graphically represent the correspondence between host and virus trees were created using the R packages phytools (1.0–3) (Revell 2012) and ape (v5.6–2) (Paradis and Schliep 2019). The virus phylogenies used in the co-phylogenies were constructed as described earlier. The relative frequencies of cross-species transmission versus virus–host co-divergence were quantified using the Jane package, which employs a maximum parsimony approach to establish the best ‘map’ of the virus phylogeny onto the host phylogeny (Conow et al. 2010). The cost of duplication, host jumping, and extinction event types were set to 1.0, while the cost of virus–host co-divergence was set to zero as it was considered the null event. The number of generations and the population size were set to 100. Jane was chosen over its successor eMPRess (Santichaivekin et al. 2020), as it allows a virus to be associated with multiple host species and handles polytomies (Santichaivekin et al. 2020). For a multi-host virus, each association was represented as a polytomy in the virus phylogeny. A co-phylogenetic analysis of the genus *Flavivirus* was not conducted as vector-borne viruses with both invertebrate and invertebrate hosts are problematic to incorporate into analyses of this kind.

## 3. Results

**Figure 1.**
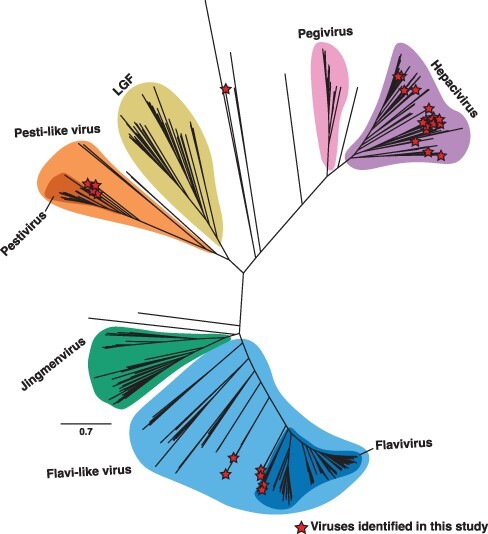
Phylogeny of the *Flaviviridae*. Unrooted maximum likelihood phylogenetic tree of the flavivirid sequences based on the conserved amino acid in the RdRp (NS5). All branches are scaled according to the number of amino acid substitutions per site. Established genera and notable clades that are yet to be ratified by ICTV are highlighted. Novel virus sequences identified in this study are displayed with a red star. LGF refers to the ‘large genome flaviviruses’.

Screening of transcriptomes revealed the presence of flavivirid-like sequences in 154 sequencing libraries within the SRA and TSA databases as well as one newly generated sequencing library from tunicates. The assembly and mining of these sequencing libraries identified 32 novel virus-like sequences, which were subsequently assigned as hepaci-like (20), flavivirus-like (7), pesti-like (4), and unclassified flavivirial-like sequences (1) (Table 1, Fig. 1). These virus-like sequences were predominately found in metazoan transcriptomes belonging to aquatic species (amphibians, bony fish, cnidarians, comb jellies, crustaceans, and hemichordates), although some were also found in land-dwelling vertebrates (birds, primates, and rodents). One virus-like sequence was assembled from a non-metazoan, alveolate library. No pegi-like virus sequences were found. We now examine each genus in turn.

**Table 1.** Summary table of the viruses discovered in this study.

Virus name	Abbreviation	Virus classification	Sample organism	SRA/TSA	Sequence length (bp)	% Std abundance	Closest Blast Hit (BLASTx)	% Ident (BLASTx)	*E*-value (BLASTx)	Tissue	Reference
African cichlid flavi-like virus	AfciFV	Flavi-like	Cyprichromis microlepidotus	SRR9688165	10,364	0.01	Salmon flavivirus (QJU12405.1)	38	0	Lower pharyngeal jaw	El Taher et al. (2021)
Chowder bay tunicate–associated flavi-like virus	CbtuFV	Flavi-like	*B. leachii*	PRJEB57836	10,455	0.001	Flavivirus sp.(QOQ37358.1)	29	1.00E−80	Whole body	NA
Cigar comb jelly flavi-like virus	CcjeFV	Flavi-like	*Beroe forskalii*	TSA:GHXY01073473.1	346	NA	Salmon flavivirus (QJU12405.1)	36	2.77E−07	Whole body	Schultz et al. (2020)
Cigar comb jelly flavi-like virus	CcjeFV	Flavi-like	*B. forskalii*	TSA:GHXY01284671.1	226	NA	Salmon flavivirus (QJU12405.1)	51	2.66E−29	Whole body	Schultz et al. (2020)
Cnidaria flavivirus	CnidFV	Flavivirus	*Eunicea flexuosa*	SRR12876665	10,729	0.004	Kadam virus (YP 009345035.1)	28	2.47E−312	Mixed	Prada and Hellberg (2021)
Harrimaniidae flavivirus	HarFV	Flavivirus	Harrimaniidae sp	SRR1695462	10,951	0.003	Rio Bravo virus (NP 620,044.1)	28	2.89E−278	Mixed	Cannon et al. (2014)
Photeros flavivirus	PhoFV	Flavivirus	*Photeros* sp	SRR10811635	11,290	0.003	Crangon crangon flavivirus (QCH00713.1)	38	0	Whole body	Hensley et al. (2019)
Sea-firefly flavivirus	SefiFV	Flavivirus	*Vargula hilgendorfii*	DRR171178	10,930	0.016	Crangon crangon flavivirus (QCH00713.1)	37	0	Whole body	Bessho-Uehara et al. (2020)
Brown Julie hepacivirus	BrjuHV	Hepacivirus	*Julidochromis dickfeldi*	SRR9688628	2160	0.0007	Guangxi houndshark hepacivirus (AVM87259.1)	41	1.08E−140	Liver	El Taher et al. (2021)
Brown Julie hepacivirus	BrjuHV	Hepacivirus	*J. dickfeldi*	SRR9688628	2990	0.001	Rodent hepacivirus (AGC52826.1)	43	1.08E−111	Liver	El Taher et al. (2021)
Cape dune mole-rat hepacivirus	CdmrHV	Hepacivirus	*Bathyergus suillus*	SRR2141210	6224	0.002	Bat hepacivirus (QQM18105.1)	42	0	Mixed	Davies et al. (2015)
Catfish hepacivirus	CatfHV	Hepacivirus	*G. macromaculatus*	SRR6426015	9865	0.005	Guangxi houndshark hepacivirus (AVM87259.1)	32	0	Liver	Jiang et al. (2019)
Caudopunctatus cichlid hepacivirus	CaciHV	Hepacivirus	*Neolamprologus toae*	SRR9680779	7165	0.009	Guangxi houndshark hepacivirus (AVM87256.1)	34	4.49E−284	Liver	El Taher et al. (2021)
Caudopunctatus cichlid hepacivirus	CaciHV	Hepacivirus	*N. toae*	SRR9680779	2385	0.003	Rodent hepacivirus (AGC52826.1)	43	2.96E−116	Liver	El Taher et al. (2021)
Crab-eating macaque hepacivirus	CremHV	Hepacivirus	*Macaca fascicularis*	SRR6032465	9855	0.031	Guereza hepacivirus (YP 009325369.1)	57	0	Whole blood	Speranza et al. (2018)
Cyprichromis hepacivirus	CyprHV	Hepacivirus	*Cyprichromis* sp. ‘dwarf jumbo’ AB-2019	SRR9688149	8678	0.012	Guangxi houndshark hepacivirus (AVM87256.1)	33	0	Liver	El Taher et al. (2021)
Dewindti cichlid hepacivirus	DeciHV	Hepacivirus	*Aulonocranus dewindti*	SRR9689044	1583	0.0009	Rodent hepacvirus (ATP66829.1)	50	5.08E−127	Liver	El Taher et al. (2021)
Dewindti cichlid hepacivirus	DeciHV	Hepacivirus	*A. dewindti*	SRR9689044	1345	0.0005	Hepacivirus L (YP 009679023.1)	44	4.95E−99	Liver	El Taher et al. (2021)
Featherfin cichlid hepacivirus	FeciHV	Hepacivirus	*Cyathopharynx furcifer*	SRR9680031	1070	0.0004	Nanhai ghost shark hepacivirus 1 (AVM87556.1)	44	3.73E−71	Liver	El Taher et al. (2021)
Freshwater butterflyfish hepacivirus	FrbuHV	Hepacivirus	*Pantodon buchholzi*	SRR5997808	8422	0.004	Nanhai ghost shark hepacivirus 1 (AVM87556.1)	29	4.40E−275	Viscera mixture	Sun et al. (2016)
Gold head compressiceps hepacivirus	GhcoHV	Hepacivirus	*Altolamprologus compressiceps*	SRR9688991	2553	0.002	Guangxi houndshark hepacivirus (AVM87259.1)	43	1.00E−176	Liver	El Taher et al. (2021)
Gold head compressiceps hepacivirus	GhcoHV	Hepacivirus	*A. compressiceps*	SRR9688991	1603	0.002	Hepacivirus L (YP 009679023.1)	43	3.59E−97	Liver	El Taher et al. (2021)
Greater mouse-eared bat hepacivirus	GmebHV	Hepacivirus	*Myotis myotis*	SRR2063918	3300	0.046	Rodent hepacivirus (QLM02863.1)	71	0	Pharyngeal and anal swab	Wu et al. (2012)
Greater mouse-eared bat hepacivirus	GmebHV	Hepacivirus	*M. myotis*	SRR2063918	2981	0.046	Rodent hepacivirus (QLM02863.1)	63	0	Pharyngeal and anal swab	Wu et al. (2012)
Neolamprologus buescheri hepacivirus	NebuHV	Hepacivirus	*N. buescheri*	SRR9681059	2105	0.002	Wenling shark virus (YP 009179227.1)	45	4.57E−74	Liver	El Taher et al. (2021)
Neolamprologus buescheri hepacivirus	NebuHV	Hepacivirus	*N. buescheri*	SRR9681059	2294	0.002	Rodent hepacvirus (ATP66829.1)	40	1.32E−144	Liver	El Taher et al. (2021)
Neolamprologus savoryi hepacivirus	NesaHV	Hepacivirus	*N. savoryi*	SRR9680861	5451	0.007	Wenling shark virus (YP 009179227.1)	45	2.95E−93	Liver	El Taher et al. (2021)
Neolamprologus savoryi hepacivirus	NesaHV	Hepacivirus	*N. savoryi*	SRR9680861	1223	0.001	Wenling shark virus (YP 009179227.1)	34	9.32E−231	Liver	El Taher et al. (2021)
Northern treeshrew hepacivirus	NotrHV	Hepacivirus	*Tupaia belangeri*	DRR155087	9208	0.00003	Hepacivirus P (YP 009553586.1)	56	0	Liver	Sanada et al. (2019)
Variabilichromis moorii hepacivirus	VamoHV	Hepacivirus	*V. moorii*	SRR9689131	4358	0.006	Guangxi houndshark hepacivirus (AVM87259.1)	36	1.82E−231	Liver	El Taher et al. (2021)
*Neolamprologus savoryi hepacivirus*	VamoHV	Hepacivirus	*V. moorii*	SRR9689131	2857	0.004	Rodent hepacivirus (AGC52826.1)	44	4.71E−113	Liver	El Taher et al. (2021)
Viviparous lizard hepacivirus	ViliHV	Hepacivirus	*Zootoca vivipara*	SRR11262332	11,862	0.001	Duck hepacivirus (QKT21547.1)	27	9.26E−263	Whole body	Yurchenko, Recknagel, and Elmer (2020)
White sucker hepacivirus	WhsuHV	Hepacivirus	*Catostomus commersonii*	SRR2912516	10,120	0.001	Guangxi houndshark hepacivirus (AVM87256.1)	31	0	Liver	Hahn et al. (2016)
Xenotilapia hepacivirus	XenoHV	Hepacivirus	*Xenotilapia* sp. ‘spilopterus north’ AB-2019	SRR9670143	2596	0.003	Rodent hepacivirus (QLM02868.1)	40	3.32E−118	Liver	El Taher et al. (2021)
Xenotilapia hepacivirus	XenoHV	Hepacivirus	*Xenotilapia* sp. ‘spilopterus north’ AB-2019	SRR9670143	4117	0.002	Guangxi houndshark hepacivirus (AVM87256.1)	35	6.73E−211	Liver	El Taher et al. (2021)
Zebra finch hepacivirus 1	ZefiHV1	Hepacivirus	*Taeniopygia guttata*	SRR7031415	2262	0.0001	Duck hepacivirus (QDF44087.1)	52	2.74E−180	Liver	NA
Zebra finch hepacivirus 1	ZefiHV1	Hepacivirus	*T. guttata*	SRR7031415	3396	0.0001	Jogalong virus (QHD25538.1)	49	0	Ovary	NA
Zebra finch hepacivirus 2	ZefiHV2	Hepacivirus	*T. guttata*	SRR6896649	1245	0.0002	Jogalong virus (QHD25538.1)	51	3.85E−67	Liver	Biederman et al. (2018)
Zebra finch hepacivirus 2	ZefiHV2	Hepacivirus	*T. guttata*	SRR6896649	1842	0.0001	Jogalong virus (QHD25538.1)	53	1.64E−209	Ovary	Biederman et al. (2018)
Cayenne caecilian pestivirus	CacaPV	Pestivirus	*Typhlonectes compressicauda*	SRR5591415, SRR5591441	1391	0.0000	Bovine viral diarrhoea virus 2 (AAC64056.1)	58	1.59E−128	Kidney	Torres-Sánchez et al. (2019)
Frog pestivirus	FrogPV	Pestivirus	*L. catesbeianus*	SRR5810423	15,334	0.012	Classical swine fever virus (NP 777,506)	28	1.35E−256	Ventral skin	Eskew et al. (2018)
Glass knifefish pestivirus	GlknPV	Pestivirus	*Eigenmannia virescens*	SRR6675258	14,199	0.013	Pronghorn antelope pestivirus (YP 009026415.1)	29	4.28E−232	Muscle	Thompson et al. (2018)
Transcaucasian sand viper pestivirus	TrsaPV	Pestivirus	*Vipera transcaucasiana*	SRR12802473	3054	0.0001	Bamboo rat pestivirus (UMO75502.1)	25	6.51E−50	Venom gland	Xie et al. (2022)
Transcaucasian sand viper pestivirus	TrsaPV	Pestivirus	*V. transcaucasiana*	SRR12802473	2120	0.0001	Atypical porcine pestivirus (QBJ01589.1)	53	1.65E−188	Venom gland	Xie et al. (2022)
Digyalum oweni-associated virus	DiowV	Unclassifed pesti-like virus	*Digyalum oweni*	SRR9888042	3715	0.0003	Xinzhou spider virus 3 (YP 009254746.1)	24	4.09E−13	Individual gut cell	Janouškovec et al. (2019)
Digyalum oweni-associated virus	DiowV	Unclassifed pesti-like virus	*Digyalum oweni*	SRR9888042	4594	0.0002	Soybean thrips virus 4 (QPZ88419.1)	25	8.94E−15	Individual gut cell	Janouškovec et al. (2019)

### 3.1 Genus *Flavivirus*

**Figure 2.**
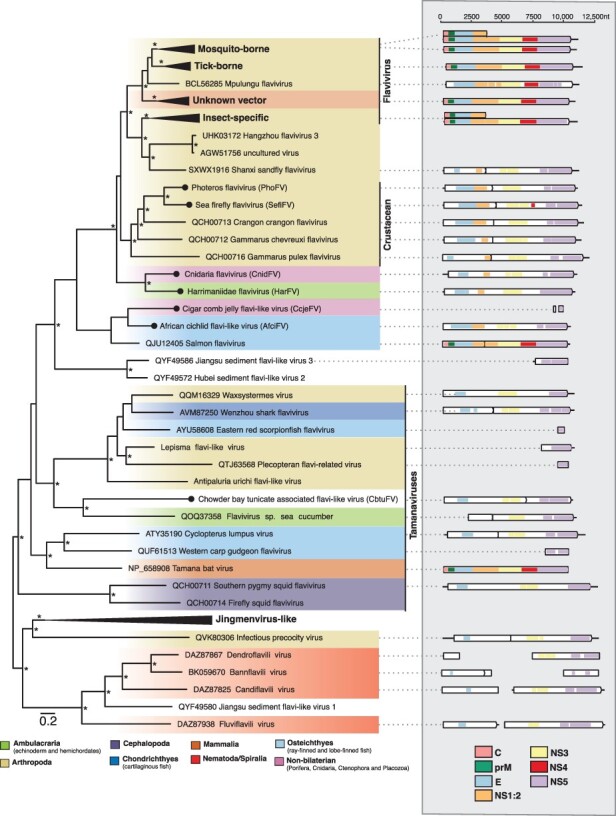
Phylogenetic relationships of the flavi-like viruses identified in this study. (Left) Phylogenetic relationships of the flavi- and jingmenviruses. ML phylogenetic trees based on the conserved amino acid in the RdRp (NS5) show the topological position of virus-like sequences discovered in this study (black circles) in the context of their closest relatives. Branches are highlighted to represent host clade (Ambulacraria = green, Arthropoda = khaki, Cephalopoda = purple, Chondrichthyes = light blue, Mammalia = orange, Nematoda/Spiralia = red, Osteichthyes = dark blue, non-bilaterian = light purple). All branches are scaled to the number of amino acid substitutions per site, and trees were midpoint rooted for clarity only. An asterisk indicates node support where SH-aLRT ≥ 80 per cent and UFboot ≥ 95 per cent. (Right) Genomic organization of the virus sequences identified in this study and representative species used in the phylogeny. The data underlying this figure and the definitions of acronyms used are presented in Supplementary Table S2.

We identified seven putative flavi-like virus sequences, including cnidaria flavivirus (CnidFV) and cigar comb jelly flavi-like virus (CcjeFV) in libraries of the early diverging metazoan phyla Cnidaria and Ctenophora, harrimaniidae flavivirus (HarFV) in an acorn worm (Enteropneusta), photeros flavivirus (PhoFV) and sea-firefly flavivirus (SefiFV) in marine ostracods, Chowder Bay tunicate–associated flavivirus in tunicates (CbtuFV), and African cichlid flavivirus (AfciFV) in a cichlid fish (Fig. 2). For all but one of these sequences (CcjeFV), complete genome sequences ranging in length from 10,364 to 11,290 nucleotides were assembled. CcjeFV consists of two partial RdRp fragments, 346 and 226 bp in length.

A range of genome structures was observed and found to be largely consistent with those found in this genus. For example, PhoFV and SefiFV, like the other viruses identified in marine crustaceans, are predicted to contain a programmed −1 ribosomal frameshift on a ‘slippery’ heptanucleotide sequence downstream of the NS1 region (Rhys, Sassan, and Williams 2019) (Fig. 2, Supplementary Fig. S1). However, CbtuFV was predicted to contain two ORFs, with the NS4/5 region encoded on the second ORF, although no ‘slippery’ heptanucleotide motifs could be detected (Fig. 2). The remaining full-length sequences were predicted to contain a single ORF. Virus domains consistent with this genus were detected across all sequences (Fig. 2).

Phylogenetic analyses of the conserved NS5 region place the ostracod sequences (PhoFV and SefiFV) within a larger diversity of marine crustacean flaviviruses. Two sequences, CnidFV and HarFV, fell basal to all classified members of the genus *Flavivirus* along with the crustacean flaviviruses (Fig. 2). Notably, these sequences appear closer in phylogenetic position and amino acid identity to tick, insect-specific, and crustacean flaviviruses than those viruses in the more divergent tamanavirus clade. The flavivirus-derived EVEs identified in the Cnidaria fell into approximately the same phylogenetic location as CnidFV and SefiFV (Supplementary Fig. S2). CcjeFV and AfciFV were placed phylogenetically with salmon flavivirus (QJU12405.1), although unlike salmon flavivirus, AfciFV consists of a single ORF.

### 3.2 Genus *Pestivirus*

**Figure 3.**
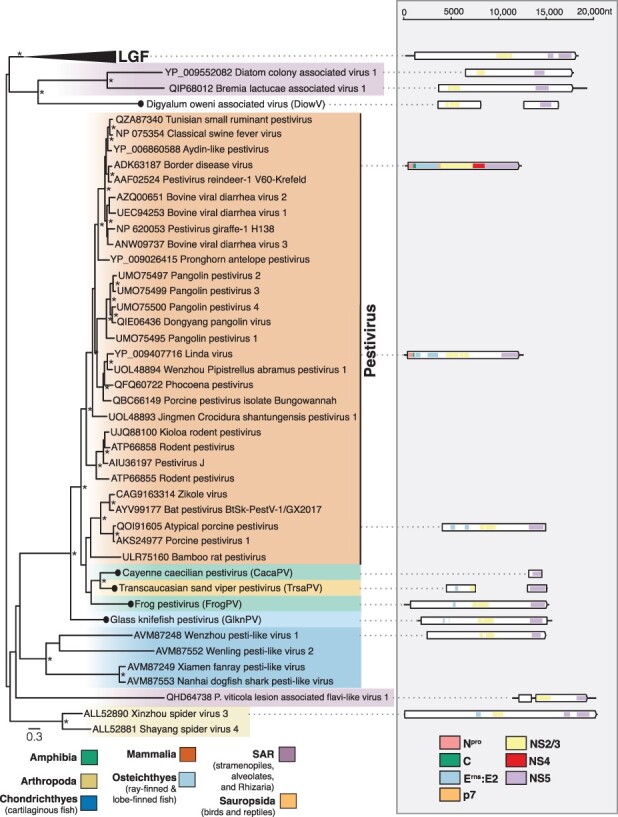
Phylogenetic relationships of the pesti-like viruses identified in this study. (Left) Phylogenetic relationships of the pestiviruses and unclassified relatives. ML phylogenetic trees based on the conserved amino acid in the RdRp (NS5) show the topological position of virus-like sequences discovered in this study (black circles) in the context of their closest relatives. The colour scheme is as found in Fig. 2, with the following exceptions, Amphibia = green, Sauropsida = light orange, SAR = light purple. All branches are scaled to the number of amino acid substitutions per site, and trees were midpoint rooted for clarity only. An asterisk indicates node support where SH-aLRT ≥ 80 per cent and UFboot ≥ 95 per cent. LGF refers to the ‘large genome flaviviruses’. Non-novel sequences without NCBI accession were obtained from Wu et al. (2020). (Right) Genomic organization of the virus sequences identified in this study and representative species used in the phylogeny. The data underlying this figure and the definitions of acronyms used are presented in Supplementary Table S2.

We identified four pesti-like virus sequences in amphibians, reptiles, and bony fish (Table 1). Two full genomes—glass knifefish pestivirus (GlknPV) and frog pestivirus (FrogPV)—were recovered, ranging from 14,199 to 15,334bp in length, in addition to two partial genomes, Transcaucasian sand viper pestivirus (FrogPV) and Cayenne caecilian pestivirus (CacaPV) (Fig. 3). These sequences exhibit more sequence similarity with mammalian pestiviruses than those associated with cartilaginous fish, with an average of 28 per cent versus 24 per cent amino acid identity across the complete polyprotein. This is reflected in the phylogenetic positioning of the novel pesti-like viruses based on the conserved NS5 region (Fig. 3). The newly identified reptile and amphibian pesti-like virus sequences, FrogPV and CacaPV, form a sister group to those found in rodents, bats, and pigs, while the sequence discovered in fish, GlknPV, fell basal to this group but remained as a sister group to those viruses from cartilaginous fish (Shi et al. 2018). The topology of the pestivirus phylogeny varied depending on whether the NS3 or NS5 domains were used in the analysis. In particular, FrogPV formed a sister lineage to the known pestiviruses in a phylogeny based on the NS3 region (Fig. 3, Supplementary Fig. S3).

### 3.3 Genus *Hepacivirus*

**Figure 4.**
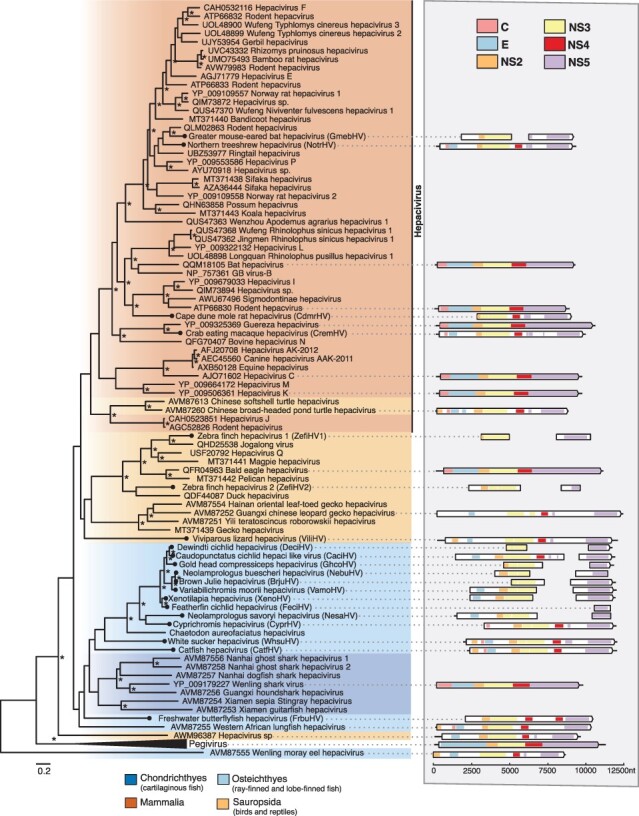
Phylogenetic relationships of the hepaciviruses viruses identified in this study. (Left) Phylogenetic relationships of the ‘pegi-hepaci’ clade. ML phylogenetic trees based on the conserved amino acid in the RdRp (NS5) show the topological position of virus-like sequences discovered in this study (black circles) in the context of their closest relatives. The colour scheme is as found in Fig. 2, with the following exception, Sauropsida = light orange. All branches are scaled to the number of amino acid substitutions per site, and trees were midpoint rooted for clarity only. An asterisk indicates node support where SH-aLRT ≥ 80 per cent and UFboot ≥ 95 per cent. (Right) Genomic organization of the virus sequences identified in this study and representative species used in the phylogeny. The data underlying this figure and the definitions of acronyms used are presented in Supplementary Table S2.

We identified 20 novel hepacivirus sequences, of which 14 were found in ray-finned fish (Actinopterygii), expanding on the two hepaciviruses previously identified in this group (Fig. 4). The remaining sequences (*n* = 6) add to the known diversity of bat, avian, primate, rodent, and treeshrew hepaciviruses (Fig. 4). Of the novel hepaciviruses, five complete genomes were assembled, ranging from 9,208 to 11,862bp in length (Fig. 4). Partial genome sequences containing at least the NS3 and NS5 domains were assembled for the remaining sequences, with the exception of the featherfin cichlid hepacivirus, for which only the NS5 region could be assembled (Fig. 4). Of note, greater mouse-eared bat hepacivirus (GmebHV) was assembled from a library generated for the analysis of bat viromes (Wu et al. 2012) and shares 70 per cent amino acid identity with rodent hepacivirus (QLM02863.1).

### 3.4 An unclassified flavivirid-like virus

In addition to the viruses that fell within established genera, we identified a partial flavi-like virus sequence termed digyalum oweni-associated virus (DiowV) in *D. oweni*, a species of parasitic protist belonging to the phylum Apicomplexa. Two contigs were assembled from this library, 3689 and 4577 bp in length and predicted to contain the NS3 and NS5 domains, respectively (Fig. 3). DiowV shares the greatest amino acid identity (24 per cent) with the Xinzhou spider virus 3 (YP_009254746) among other large genome flaviviruses (LGF). When included in the ‘pesti-LGF’ tree, DiowV, along with diatom colony–associated virus 1 (YP_009552082) and bremia lactucae–associated virus 1 (QIP68012), forms a sister group to the LGF. However, in the family-wide tree, these sequences, along with Snake River alfalfa virus (ON669064), fall outside of the ‘pesti-LGF’ lineage and basal to the ‘pegi-hepaci’ group, although these branches receive poor bootstrap support (Fig. 1).

### 3.5 Genetic composition of sequencing libraries

**Figure 5.**
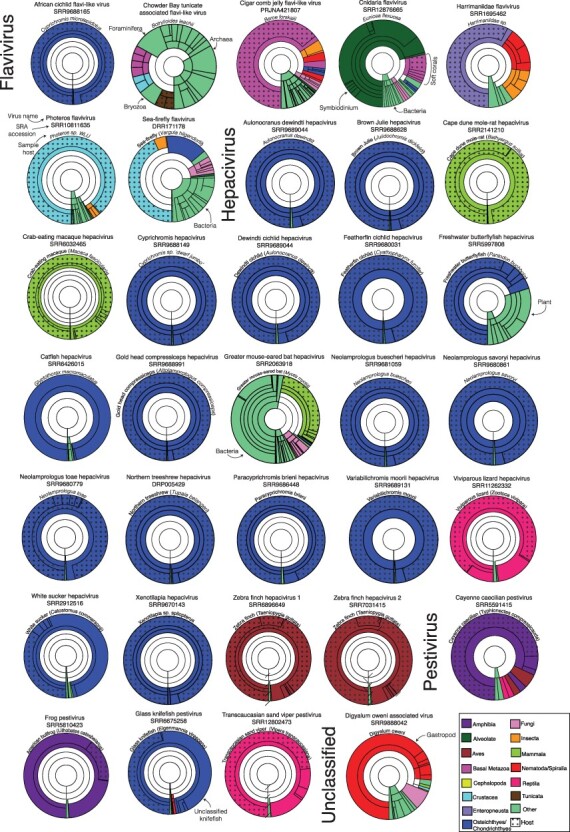
Taxonomic assignments of contigs in sequencing libraries. Each Krona graph illustrates the relative abundance of taxa in a metatranscriptome at varying taxonomic levels. For clarity, a maximum depth of five taxonomic levels was chosen for each graph. The library SRA accession number, host species, and the corresponding virus of interest are annotated above each graph. Segments are highlighted based on the species’ taxonomic grouping. Dots have been used to signify where contigs have been taxonomically assigned within the same family as the host species. Contigs without any matches in the database are not shown.

Metagenomic sequencing libraries are often comprised of organisms in addition to the target host, which can complicate virus–host assignment. To quantify the composition of these libraries and improve virus–host assignments, we utilized the KMA and CCMetagen tools (Fig. 5). For 20 of the libraries, over 80 per cent of eukaryotic contigs were assigned to the expected target host of the sequencing library (median, 90 per cent; range, 0–98 per cent). In the case of the *E. flexuosa* (family *Plexauridae*) library in which CnidFV was assembled, a genus of unicellular microalgae, *Symbiodinium* (phylum Dinoflagellata), represented 64 per cent of all contigs (Fig. 5). In this library, soft corals (order Alcyonacea, phylum Cnidaria), which include *E. flexuosa*, represented 63 per cent of metazoan abundance, while tunicates and bony fish represented 13 and 10 per cent of abundance, respectively. Despite *Plexauridae* comprising 60 per cent of cnidarian abundance, other soft coral families were also detected, including the *Ellisellidae, Nephtheidae, Acanthogorgiidae*, and *Nidaliidae*, each representing ∼10 per cent of cnidarian abundance. Likewise, the tunicate library from which CbtuFV was assembled comprised reads belonging to various marine organisms, including Bryozoa, Cnidaria, and crustaceans, representing an average of 8 per cent abundance each.

Contigs belonging to catfish (order Siluriformes) comprised 95 per cent of the *Glyptothorax macromaculatus* library from which catfish hepacivirus (CatfHV) was assembled, although it is uncertain to which family of catfish this sample belonged. Likewise, the American bullfrog (*Lithobates catesbeianus)* transcriptome comprised 60 per cent contigs associated with fork-tongued frogs (*Dicroglossidae*) and 17 per cent associated with true frogs (*Ranidae*), including *L. catesbeianus*. No host-associated contigs were detected in the *D. oweni* library in which DiowV was assembled. Instead, 64 per cent of the library is composed of contigs associated with marine gastropod molluscs.

### 3.6 Long-term virus–host evolutionary relationships

**Figure 6.**
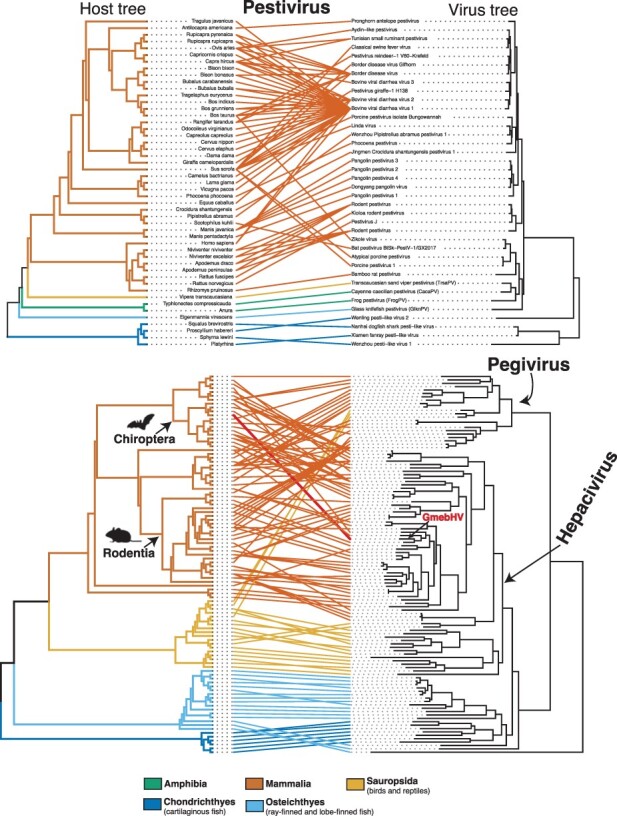
Tanglegram of rooted phylogenetic trees for representative virus groups and their hosts. Branches of the host tree (left) and lines are coloured to represent the host clade. The colour scheme is as found in Fig. 2, with the following exceptions, Amphibia = green, Sauropsida = light orange. All branches on the virus tree are scaled to the number of amino acid substitutions per site, and both trees were midpoint rooted for clarity only. Greater mouse-eared bat hepacivirus (GmebHV) is highlighted in red. Images were obtained from http://phylopic.org under Public Domain Dedication. Supplementary Fig. S5 provides the names of the hosts and viruses for the ‘pegi-hepaci’ co-phylogeny.

To examine the frequency of four macroevolutionary events (i.e. co-divergence, duplication, host-switching, and extinction) among the *Flaviviridae*, we estimated co-phylogenies to quantify the evolutionary relationship between the ‘pegi-hepaci’ and pestivirus clades and their hosts (Fig. 6; members of the genus *Flavivirus* were excluded because of the high frequency of vector-borne viruses). In accordance with earlier studies (Geoghegan, Duchêne, and Holmes 2017), this analysis revealed that cross-species transmission was the most common evolutionary event across the ‘pegi-hepaci’ and pestivirus clades, representing 65 and 71 per cent of events, respectively (Supplementary Fig. S4). Two viruses, GmebHV and freshwater butterflyfish hepacivirus (FrbuHV) identified in this study, present notable exceptions (Fig. 6). GmebHV is distinct from known bat hepaciviruses (Hepacivirus K, Hepacivirus L, and Hepacivirus M), and instead groups with those viruses found in rodents, shrews, sloths, and raccoons (Fig. 4). FrbuHV, along with Western African lungfish hepacivirus and Wenling moray eel hepacivirus, fell basal to those viruses identified in cartilaginous fish.

Importantly, despite the widespread occurrence of cross-species transmission, virus–host co-divergence was also predicted to have occurred relatively frequently across the ‘pegi-hepaci’ and pestivirus clades, representing 22 and 23 per cent of all events, respectively. For these groups, duplication events were more uncommon, representing 10 and 6 per cent of total events (Supplementary Fig. S4). Extinction events were rarely predicted, representing 4 per cent of events in the ‘pegi-hepaci’ clade, while no extinction events were detected in the Pestivirus co-phylogeny.

## 4. Discussion

Through transcriptome mining, we identified 32 novel flavivirid sequences across the Metazoa, including the first flavivirus-like sequences in non-bilaterians, pestivirus-like sequences in amphibians, reptiles, and bony fish, as well as a range of vertebrate hepaciviruses. Hence, this work provides further evidence of the long-term associations between the *Flaviviridae* and Metazoa and highlights the vast number of viruses that remain undiscovered.

The Cnidaria are a primitive and basal phylum of Metazoa. Based on the identification of a flavivirus-like sequence in a cnidarian sample (CnidFV), we suggest that the origins of this group of viruses likely extend much further back in time than previous estimates and are closer to the emergence of the metazoans 750–800 million years ago (Erwin 2015). This conclusion is supported by the earlier finding of a flavivirus-derived EVE in the Cnidaria (Bamford et al. 2022). Notably, CnidFV and the cnidarian EVE are more closely related to members of the genus *Flavivirus* than are the tamana/flavi-like viruses, suggesting that these groups, including the jingmenviruses, are evolutionarily distinct (Bamford et al. 2022). As such, we suggest that the tamana/flavi-like viruses should be given a distinct taxonomic classification within the *Flaviviridae*. However, it is clear that it is difficult to fully resolve the evolutionary history of the flavivirids with our current understanding of their diversity, although it appears that the origins of this group lie in aquatic environments (Lensink, Yiqiao, and Lequime 2022).

It is important to note that host assignment of the non-bilaterian flaviviruses is tentative as these sequences are extremely divergent and have only rarely been sampled. Due to the detection of several cnidarian species in addition to the target species, the octocoral *E. flexuosa* in library SRR12876665, we have assigned the resulting virus sequence as cnidaria flavivirus (CnidFV). The high abundance of *Symbiodinium* in this library is unsurprising given that the octocoral-*Symbiodinium* mutualism is well known (van de Water, Allemand, and Ferrier-Pagès 2018). However, the phylogenetic placement of this virus with those found in a marine acorn worm suggests that it is more likely associated with *E. flexuosa* than *Symbiodinium*. While CnidFV and the peach blossom jellyfish EVE are relatively closely related to each other, there is substantial genetic divergence between these sequences. This has been previously observed with crocidura pestivirus and a Crocidura EVE and may reflect divergent evolution since the historic endogenization event (Li et al. 2022).

The discovery of Wenzhou pesti-like virus 1, Wenling pesti-like virus 2, Xiamen fanray pesti-like virus, and Nanhai dogfish shark pesti-like virus in cartilaginous fish marked the expansion of the pestiviruses from warm-blooded mammals to basal vertebrate species, suggesting that these viruses infect a range of vertebrate lineages (Shi et al. 2018). For the first time, we identified pesti-like viruses in reptiles, amphibians, and bony fish, extending the host range of these viruses to encompass all vertebrate classes with the exception of Aves. The deep evolutionary association between pestiviruses and vertebrates is further reflected in the clear pattern of pestivirus–host co-divergence among the viruses identified in this study. As a result, we anticipate that novel pestiviruses will be found infecting a wider diversity of vertebrates and that their known host range largely reflects where sampling efforts have been directed to date. Additionally, frog pestivirus was identified in the ventral skin of the American bullfrog, although other species of frog were detected in this library. Within the study in which this library was generated, the American bullfrog appeared resistant to the fungal pathogen *Batrachochytrium dendrobatidis* (Bd) (Eskew et al. 2018). Co-infection with Bd and ranaviruses is frequently observed in frogs, but whether the interactions between these pathogens are antagonistic or facilitative is currently unclear (Bosch et al. 2020). If Bd and pestiviruses are found to commonly co-infect frogs, future efforts should be directed towards studying their interactions.

We identified 20 novel hepacivirus sequences, among which a clade of cichlid-associated hepacivirus sequences is notable. This clade was derived from a study of Lake Tanganyika, a freshwater lake shared by Tanzania, the Democratic Republic of the Congo, Burundi, and Zambia that is known for its high diversity of endemic cichlid species (Koblmüller et al. 2008; El Taher et al. 2021). Importantly, the fish and reptile hepaciviruses identified in this study were predominately associated with samples of liver tissue, suggesting that hepatotropism is likely a universal feature of these viruses across vertebrates (Smith et al. 2016).

Bats and rodents harbour a large diversity of hepaciviruses and are thought to have played an important role in their global spread and broader evolutionary history (Epstein et al. 2010; Drexler et al. 2013; Kapoor et al. 2013; Quan et al. 2013; de Souza et al. 2019; Bletsa et al. 2021). We identified GmebHV, which falls within a clade of rodent, sloth, and raccoon hepaciviruses. The clear relatedness between GmebHV and rodent hepacivirus (QLM02863), combined with evidence from our co-evolutionary analyses, suggests that this sequence might represent a cross-species transmission event between bats and rodents. Similarly, ancestral state reconstructions have previously shown that cross-species transmission from rodents is likely the source of the sloth and ringtail hepaciviruses (Moreira-Soto et al. 2020; Jo et al. 2022). In this case, we cannot resolve the direction of virus transmission with any certainty or whether other species are involved.

In very broad terms, we find that the hepaci-, pesti-, and pegiviruses cluster with the phylogeny of their hosts, with the relevant frequent cross-species virus transmission events only occurring within host classes (i.e. Mammalia, Sauropsida, and Chondrichthyes). The exceptions were FrbuHV, Western African lungfish hepacivirus, and Wenling moray eel hepacivirus that fell basal to those viruses identified in cartilaginous fish (although the phylogenetic position of these viruses should be treated with caution as the relevant nodes have weak bootstrap support; Fig. 4, Fig. 6). The clear phylogenetically defined barriers between host classes may reflect differences in receptor binding and cell entry mechanisms among distantly related hosts (Parrish et al. 2008). Host ecology also likely contributes to these barriers, particularly as physical separation means that fewer cross-species virus transmission events are expected to occur between marine and land vertebrates than among land vertebrates (Luis et al. 2015; French et al. 2022). Together, this suggests that more cross-species transmission occurs among closely related hosts, which may have also resulted in the apparent loss of co-divergence signal within relatively well-sampled taxonomic groups (e.g. mammals). At deeper taxonomic levels, we found clear evidence for virus–host co-divergence, particularly in lower vertebrates, which is consistent with previous findings (Hartlage, Cullen, and Kapoor 2016; Geoghegan, Duchêne, and Holmes 2017; Shi et al. 2018; Porter et al. 2020). However, it is also apparent that the results of our co-phylogenetic analysis are influenced by the sample of virus diversity used and will likely change as more viruses are identified. In addition, virus phylogenies were estimated using RdRp (NS5) alone. It is possible that differences in the phylogenies of the entire polyprotein or NS3 region would produce different estimates of the frequencies of co-divergence and host jumping.

Wenling moray eel hepacivirus (AVM87555) forms a sister group to the ‘pegi-hepaci’ lineage, although this may be artefactual due to recombination or extreme rate variation (Porter et al. 2020). If the position of the Wenling moray eel hepacivirus is correct, this suggests that a common ancestor of the ‘pegi-hepaci’ lineage may have existed in an aquatic environment. This notion is supported by the recent finding of ‘pegi-hepaci’ derived EVE in a marine mollusc (Bamford et al. 2022). The apparent lack of pegiviruses in aquatic vertebrate and invertebrate species in this study does not equate to their absence in these organisms due to the current depth of SRA libraries available.

Another notable observation from this study was the identification of a flavivirus in non-bilaterians, which raises additional questions on the ancestral mode of flavivirus transmission. Non-bilaterians lack the circulatory system of vertebrates, suggesting that an alternative mode of cell-to-cell virus transmission may exist in these animals (Bamford et al. 2022).

In sum, through a broad-scale survey of publicly available and newly generated transcriptome data, we revealed a wide diversity of flavivirid sequences in undersampled metazoan species. In doing so, we provide additional information for an ancient origin of the flaviviruses, likely closer to the emergence of the metazoans some 750–800 million years ago, and hence for the long-term association between the *Flaviviridae* and the Metazoa as a whole.

## Supplementary Material

veac124_Supp

## Data Availability

All tunicate sequence reads are available on the NCBI SRA under BioProject PRJEB57836. All viral genomes and corresponding sequences assembled in this study have been deposited in the European Nucleotide Archive at EMBL-EBI and GenBank under BioProject PRJEB57836. The sequences, alignments, phylogenetic trees, and the custom *Flaviviridae* database generated in this study are available at https://github.com/JonathonMifsud/Transcriptome_mining_extends_the_host_range_of_the_Flaviviridae_to_non-bilaterians.
