# Supplementary material for: Transcriptome mining extends the host range of the Flaviviridae to non-bilaterians
Source: Virus Evol. 2022 Dec 26;9(1):veac124. doi: 10.1093/ve/veac124 (PMC9854234; doi:10.1093/ve/veac124)
Supplement: veac124_Supp [file veac124_supp.zip › suppl_data/Supplementary_figures.pdf]

Supplementary Material for: Transcriptome mining extends the host range of the Flaviviridae to non-bilaterians

Jonathon C.O. Mifsud[1], Vincenzo A. Costa[1], Mary E. Petrone[1], Ezequiel M. Marzinelli[2,3,4], Edward C. Holmes[1]*, Erin Harvey[1]

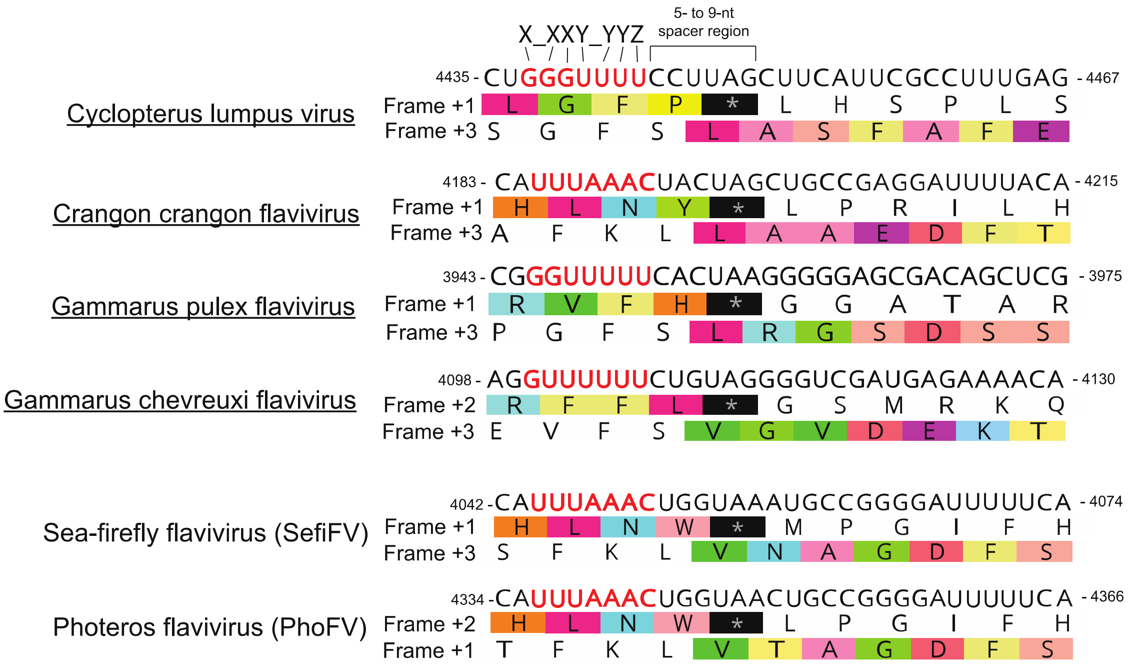

Supplementary Figure 1. Alignment of the predicted structured programmed ribosomal frameshift (−1 PRF) regions identified with reference sequences. Slippery heptanucleotide regions are highlighted in red. Predicted translated protein regions are indicated in colour.

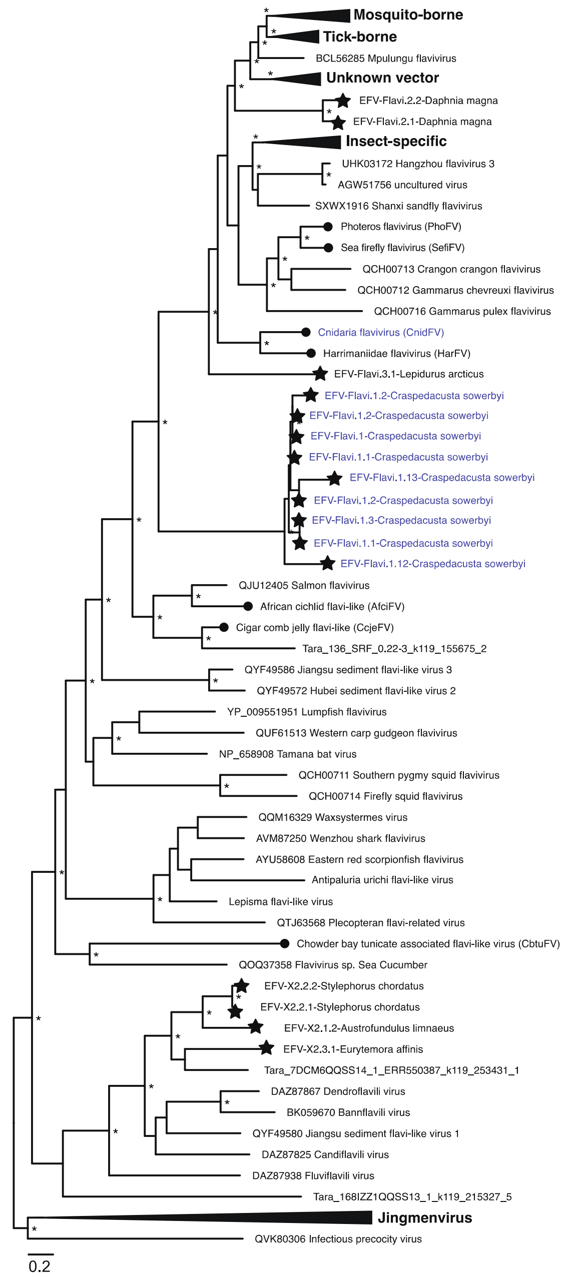

Supplementary Figure 2. Phylogenetic relationships of the flavi-like viruses and flavivirus-derived EVEs. Phylogenetic relationships of the 'flavi-jingmen' clade including the EVE identified in Bamford et al. (2022) (black stars). ML phylogenetic trees based on the conserved amino acid in the NS5 region show the topological position of virus-like sequences discovered in this study (black circles) in the context of their closest relatives. All branches are scaled to the number of amino acid substitutions per site, and trees were midpoint rooted for clarity only. An asterisk indicates node support where SH-aLRT >= 80% and UFboot >= 95%.

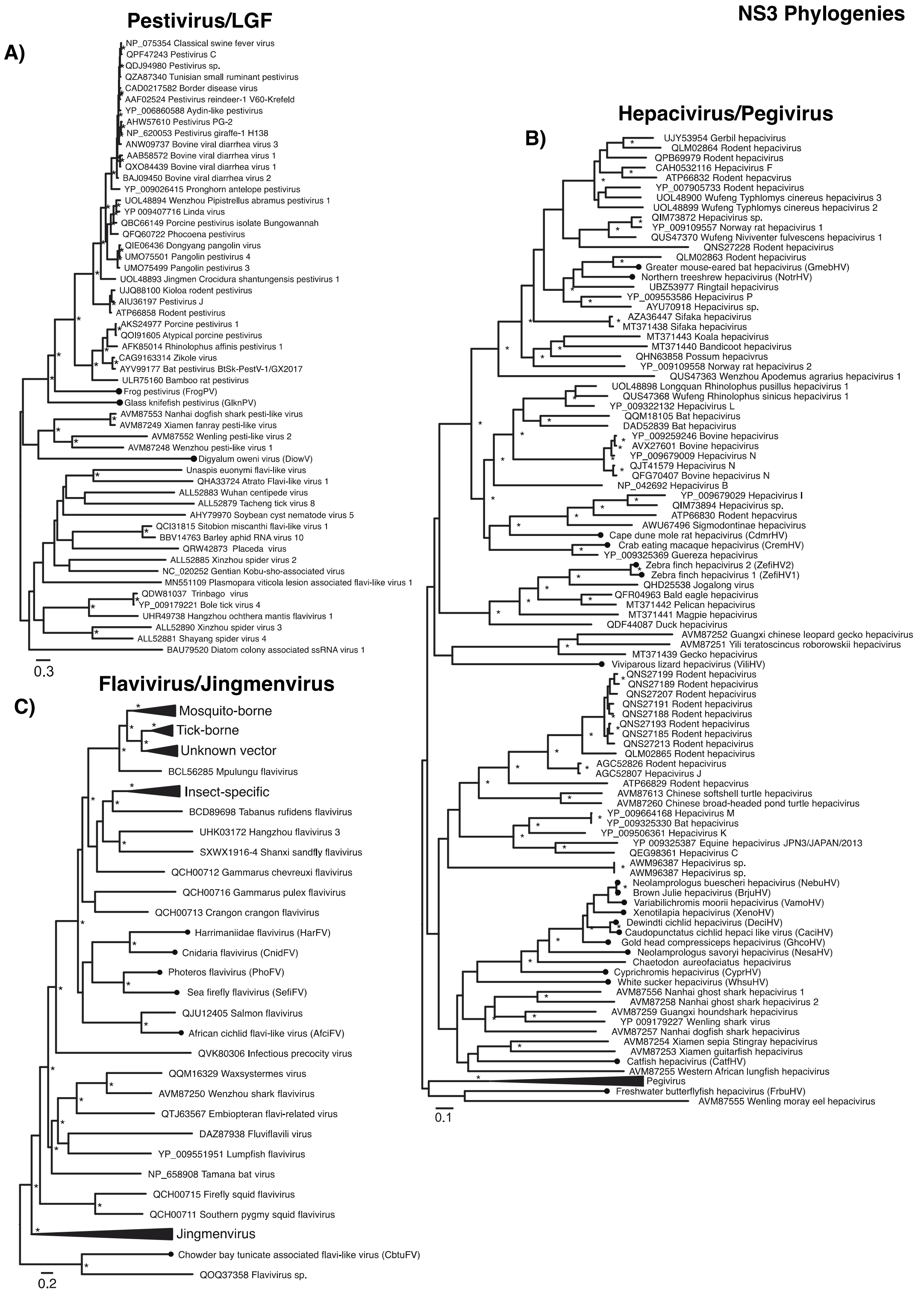

Supplementary Figure 3. Alternative phylograms based on the conserved NS3 region. Phylogenetic relationships of the ‘flavi-jingmen’, ‘pegi-hepaci’, and ‘pesti-LGF’ clades. ML phylogenetic trees based on the conserved amino acid in the NS3 region show the topological position of virus-like sequences discovered in this study (black circles) in the context of their closest relatives. All branches are scaled to the number of amino acid

substitutions per site, and trees were midpoint rooted for clarity only. An asterisk indicates node support where SH-aLRT >= 80% and UFboot >= 95%.

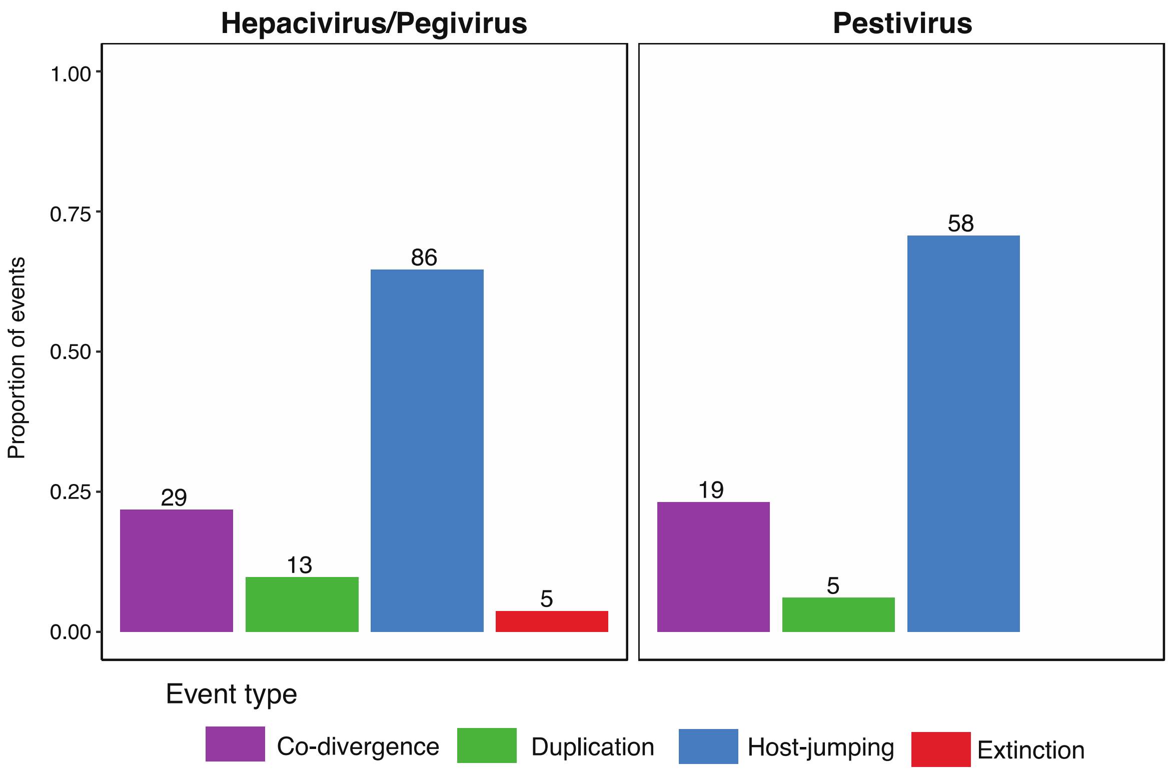

Supplementary Figure 4. Reconciliation analysis. Reconciliation analysis of select virus groups. Bar plots illustrate the range of the proportion of possible events and are coloured by event type.

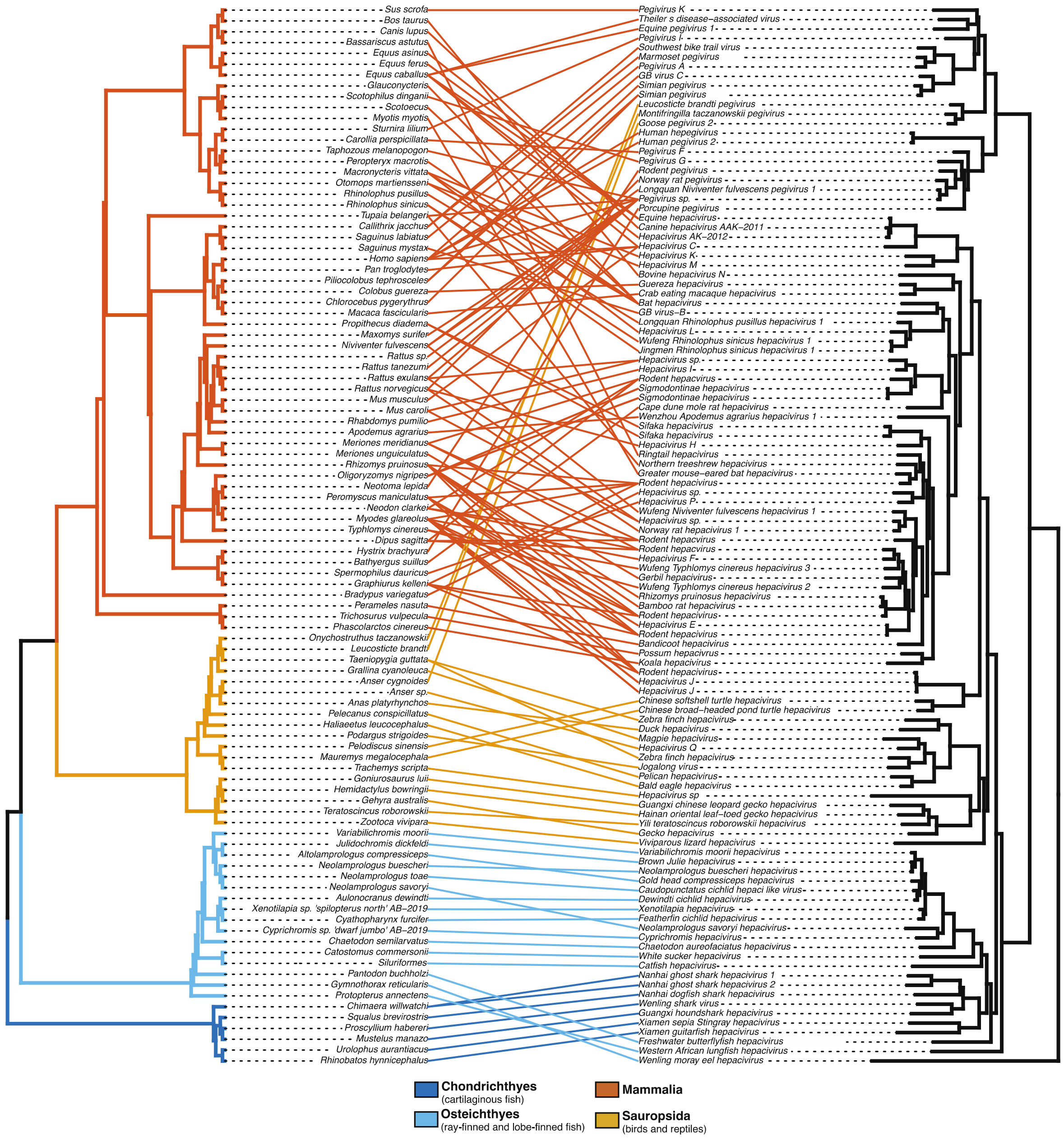

Supplementary Figure 5. Tanglegram of the 'pegi-hepaci' clade and their hosts with species labels present. Branches of the host tree (left) and lines are coloured to represent the host clade. All branches on the virus tree are scaled to the number of amino acid substitutions per site, and both trees were midpoint rooted for clarity only.
